# Maternal breastfeeding is associated with offspring microbiome diversity; a secondary analysis of the MicrobeMom randomized control trial

**DOI:** 10.3389/fmicb.2023.1154114

**Published:** 2023-08-31

**Authors:** Cara A. Yelverton, Sarah Louise Killeen, Conor Feehily, Rebecca L. Moore, Shauna L. Callaghan, Aisling A. Geraghty, David F. Byrne, Calum J. Walsh, Elaine M. Lawton, Eileen F. Murphy, Douwe Van Sinderen, Paul D. Cotter, Fionnuala M. McAuliffe

**Affiliations:** ^1^UCD Perinatal Research Centre, School of Medicine, University College Dublin, National Maternity Hospital, Dublin, Ireland; ^2^APC Microbiome Ireland, National University of Ireland, Cork, Ireland; ^3^Teagasc Food Research Centre, Moorepark, Fermoy, Cork, Ireland; ^4^UCD Institute of Food and Health, School of Agriculture and Food Science, University College Dublin, Dublin, Ireland; ^5^PrecisionBiotics Group Ltd., Cork, Ireland; ^6^School of Microbiology, University College Cork, Cork, Ireland

**Keywords:** pregnancy, infant health, microbiome, well-being, breastfeeding

## Abstract

**Background:**

Microbial dysbiosis in infancy can influence long-term health outcomes such as childhood obesity. The aim of this study is to explore relationships among maternal well-being during pregnancy, breastfeeding, and the infant gut microbiome.

**Methods:**

This is a secondary analysis of healthy pregnant women from the MicrobeMom study, a double-blind randomized control trial of maternal probiotic supplementation (*Bifidobacterium breve* 702258) versus placebo antenatally and up to 3 months postpartum. Maternal well-being was assessed using the WHO-5 well-being index at 16 weeks’ and 34 weeks’ gestation. Breastfeeding practices were recorded at discharge from hospital and at 1 month postpartum. Infant stool samples were obtained at 1 month of age. Next generation shotgun sequencing determined infant microbial diversity. Independent sample *t*-tests and Mann-Whitney *U* tests informed adjusted regression analysis, which was adjusted for delivery mode, antibiotics during delivery, maternal age and body mass index (BMI), and probiotic vs. control study group.

**Results:**

Women (*n* = 118) with at least one measure of well-being were on average 33 years (SD 3.93) of age and 25.09 kg/m^2^ (SD 3.28) BMI. Exclusive breastfeeding was initiated by 65% (*n* = 74). Any breastfeeding was continued by 69% (*n* = 81) after 1 month. In early and late pregnancy, 87% (*n* = 97/111) and 94% (*n* = 107/114) had high well-being scores. Well-being was not associated with infant microbial diversity at 1 month. In adjusted analysis, exclusive breastfeeding at discharge from hospital was associated with infant microbial beta diversity (PC2; 0.254, 95% CI 0.006, 0.038). At 1 month postpartum, any breastfeeding was associated with infant microbial alpha diversity (Shannon index; −0.241, 95% CI −0.498, −0.060) and observed species; (−0.325, 95% CI −0.307, −0.060), and infant microbial beta diversity (PC2; 0.319, 95% CI 0.013, 0.045). Exclusive breastfeeding at 1 month postpartum was associated with infant alpha diversity (Shannon index −0.364, 95% CI −0.573, −0.194; Simpson index 0.339, 95% CI 0.027, 0.091), and infant’s number of observed microbial species (−0.271, 95% CI −0.172, −0.037).

**Conclusion:**

Breastfeeding practices at 1 month postpartum were associated with lower microbial diversity and observed species in infants at 1 month postpartum, which is potentially beneficial to allow greater abundance of *Bifidobacterium*.

**Clinical trial registration:**

ISRCTN53023014.

## 1. Introduction

Colonization of the infant gut is emerging as a priority area for health research in recent years. The first 1,000 days of life are crucial for the appropriate development of the infant gut microbiome, as it undergoes significant changes with the progression from milk to solid food (Kapourchali and Cresci, 2020). It has been shown that members of the genus *Bifidobacterium* are dominant colonizers of the infant gut, and low relative abundance of this genus in early life has been associated with an increased risk of non-communicable diseases (Björkstén et al., 2001; Kalliomäki et al., 2008). In the early years, the microbiome is quite dynamic, and the early colonizers of the infant gut are believed to impose a lasting impact on host health throughout life. The gut microbiome experiences significant changes and instability in the first few years yet becomes more settled in adolescence and will remain relatively stable throughout adulthood, reflecting a development process where early life gut colonization is an important and defining event (Kumbhare et al., 2019; O’Neill et al., 2020). Research in this area is still expanding and, to gain true understanding of how these factors may influence long term offspring health, further investigations are needed.

Gut health may potentially mediate the connection between diet and mental health via the gut brain axis (Dreher, 2018). The gut brain axis is a bidirectional pathway by which the enteric and central nervous systems communicate, linking intestinal function to emotional and cognitive centers of the brain (Quigley, 2018). The gut is responsible for a large proportion of serotonin production in the body, influencing mental health, and higher microbial diversity may relate to increased serotonin production (Yano et al., 2015). Our work previously demonstrated dietary intakes to have an impact on mental health during pregnancy, and it was suggested that this is by way of the gut-brain axis (Maher et al., 2020; Yelverton et al., 2022). Maternal prenatal stress has been associated with infant intestinal microbiotia in animals (Bailey et al., 2004) and humans (Zijlmans et al., 2015). It is therefore plausible that maternal well-being may alter infant microbial diversity.

The potential for mental health to influence offspring gut health may be mediated by breastfeeding behaviors, as perinatal depression has been associated with poor breastfeeding habits (Fairlie et al., 2009). Prenatal history or history of depression was observed to negatively impact infant gut microbial diversity (Campos-Rodríguez et al., 2013; Rodriguez et al., 2021). Prenatal distress and mental ill-health have been, to some extent, associated with offspring gut health (Kang et al., 2018, 2020), however research is limited and few have investigated microbial diversity in particular. There is a paucity of data pertaining to general well-being in pregnancy and offspring microbiome diversity.

Breastfeeding is one of the most important factors for infant gut colonization. Exclusive breastfeeding is most beneficial for offspring health for numerous reasons (Pattison et al., 2019), and such benefits extend into infancy, childhood and adulthood. Human breastmilk contains numerous biologically active molecules, including microbes, that cannot be replicated in formula feed, hence the benefits are exclusive to breastmilk (Lyons et al., 2020). Multiple studies have shown an association between breastmilk and improved colonization of the infant gut microbiome (Pannaraj et al., 2017; Savage et al., 2018; Williams et al., 2019). The association with microbial diversity is less defined, but has been suggested to be an inverse relationship (Bäckhed et al., 2015; Rautava, 2016). It is important to continuously demonstrate such benefits in observational data as replication of associations is essential for confirming a true relationship that may lead to clinical trials to demonstrate causality.

This work aimed to explore the relationship between maternal well-being during pregnancy and breastfeeding practices with infant microbiome diversity at 1 month of age.

## 2. Methods

### 2.1. MicrobeMom

The MicrobeMom Study was a double-blind, randomized, placebo-controlled trial of a probiotic during pregnancy to assess its ability to transfer to the neonatal stool, an area that few studies have investigated (Moore et al., 2020). The study took place at the National Maternity Hospital, Dublin, Ireland from September 2016 to June 2019. Ethical approval was granted by the National Maternity Hospital Ethics Committee in February 2019 (EC 35.2015). The trial was registered in August 2016 with the Clinical Trial Registry (ISRCTN53023014). The probiotic bacterium used for this study was *Bifidobacterium breve* 702258. This bifidobacterial strain, referred to as the probiotic in this study, was chosen as it had previously been shown to increase stearic, arachidonic, and docosahexaenoic acid concentrations in murine liver and brain models (Swanson et al., 2020; Arabi et al., 2022). These are important conjugated linoleic acids involved in infant development and therefore this strain may have the potential to beneficially alter infant health. The trial products were provided as identical white, hydroxypropylmethylcellulose capsules, packaged by PrecisionBiotics Ltd., in an extruded aluminum tube. They were produced in their external CMO and manufactured under good manufacturing practice conditions. The probiotic underwent rigorous quality control and quality assurance assessment before it was released. Both live counts and total bacterial cell counts were performed to formulate the “probiotic” capsules to contain 10 mg of live, culturable probiotic as freeze-dried powder blended with standard excipients at a dose 1 × 109 colony forming units (CFU). Placebo capsules contained standard inactive ingredient. Participants were eligible for inclusion if they were attending the National Maternity Hospital for their antenatal care, were < 14 weeks gestation at booking visit, singleton pregnancies with no medical conditions requiring medication, no history or current diagnosis of diabetes or gestational diabetes mellitus, aged over 18 years, a pre-pregnancy BMI of 18.5–35 kg/m^2^, were not taking any probiotics and were willing to give up any probiotic containing foods, and had an adequate level of English to understand the study and give fully informed, written consent. The results of this trial demonstrated the supplemented probiotic to directly transfer from mother to infant, however it did not note any changes in microbial community diversity in infants (Moore et al., 2023). There are 118 mother-infant pairs included in this analysis based on the presence of infant microbial diversity outcomes.

### 2.2. Well-being

Well-being was measured using the World Health Organization 5-Item Well-being index (WHO-5 index). This is a validated tool that has been used to predict risk of depression in multiple populations (Sisask et al., 2008; Mortazavi et al., 2015; Broberg et al., 2021). It is a clinically pragmatic tool that has proven to be capable of identifying those at risk of depression in several populations, both pregnant and non-pregnant (Mortazavi et al., 2015; Topp et al., 2015; Rauwerda et al., 2018). At 16 weeks’ and again at 34 weeks’ gestation, participants were asked to answer the WHO-5 item well-being index. The index is a self-reported questionnaire asking participants to consider the following five questions during the preceding 2 weeks; (1) *‘I have felt cheerful and in good spirits,’* (2) *‘I have felt calm and relaxed,’* (3) *‘I have felt active and vigorous,’* (4) *‘I woke up feeling fresh and rested,’* and (5) ‘*my daily life has been filled with things that interest me*.’ Participants answered using the following six-point Likert scale ranging from “at no time” which equates to zero points to “all of the time,” equating to five points. The scores for each question are added together to yield a raw well-being score. The maximum raw score is 25, indicating the highest possible well-being. Raw scores are multiplied by four to give a percentage score. For our study, we further dichotomized participants into satisfactory well-being scores (>50%) and low well-being scores (≤50%), as this categorization has been shown to demonstrate those at risk of poor mental health outcomes (Bech et al., 2003).

### 2.3. Breastfeeding

Breastfeeding practices upon hospital discharge were recorded from hospital records as breastfeeding yes/no and exclusive breastfeeding yes/no. At the 1-month follow-up, participants were asked whether they had breastfed their child and whether it was exclusive breastfeeding or not. This was further categorized to align with breastfeeding habits recorded upon hospital discharge as any breastfeeding yes/no and exclusive breastfeeding yes/no.

### 2.4. Infant microbiome diversity

Infant stool samples were collected from baby’s nappies within 24 h of the 1-month postpartum study visit in 3 stool pots. The samples were divided appropriately between the 3 stool pots in the microbiology lab using a fume extractor. There was 1 g of infant stool in one stool pot, fixed with 7 mL of RNA*later* and frozen on site at −80°C. Within 8 weeks of freezing, the samples were transferred to Teagasc Food Research Centre for DNA and RNA extraction using the AllPrep DNA/RNA kit (Qiagen), followed by shotgun metagenomic sequencing of DNA on the Illumina NextSeq platform. All the libraries were sequenced employing the 2 × 300 bp paired end kit. The employed methods were previously described in detail (Feehily et al., 2023; Moore et al., 2023). As reported previously, metagenomic sequencing and quality filtering was performed (Feehily et al., 2023). Following quality filtering of sequenced DNA, the compositional profile of the microbiota was determined with the MetaPhlAn3 database version v30. Both alpha diversity and Beta diversity were calculated in R using vegan’s Adonis function. Alpha diversity was measured by Simpson index, Shannon index and number of observed species. The Bray–Curtis dissimilarity measure was used for Beta diversity with Principal Component Analysis (PCA). Rarefaction was performed on the infant 1-month stool samples. Only samples with more than 100,000 reads were included and the rarefaction demonstrates sufficient representation of microbial diversity.

### 2.5. Baseline demographic information

Participant demographic information was recorded at first study visit (16 weeks’ gestation) including age, height, weight, body mass index (BMI; kg/m^2^), ethnicity and education level. Pobal Haase & Pratschke Deprivation Index (HP index) was determined using participant address and is a proxy of socio-economic status based on the 2016 census in Ireland (Haase and Pratschke, 2018). Education was categorized as having achieved 3^rd^ level education (including a college, university, regional technical college, or institute of technology). Ethnicity was categorized as Caucasian or not Caucasian. Participants were asked if they were taking prenatal supplements, and this was recorded as yes/no. Alcohol and smoking habits were recorded at both 16 weeks’ and 34 weeks’ gestation. If participants noted that they had consumed alcohol or had smoked at either timepoint, they were categorized as having alcohol in pregnancy or having smoked in pregnancy, respectively. Both were then categorized as yes/no variables and the prevalence of not consuming alcohol or smoking is reported. Number of previous children was recorded, and those with no previous children were categorized as yes for first child. Delivery outcomes were recorded from hospital records, including gestational age in days, birthweight (g), if it was a Cesarean delivery or spontaneous vaginal delivery, if mothers received antibiotics in labor, infant sex, and if the infant was admitted to intensive care (NICU; Neonatal Intensive Care Unit).

### 2.6. Statistical analysis

Statistical analysis was carried out using SPSS version 26. All continuous variables were checked for normality by comparing means and medians, assessing skewness and kurtosis, and using histograms as visual aid. Non-parametric data included Pobal Haase & Pratschke Deprivation Index (HP index), Simpson index, observed species, and PC2. All other values, including the Shannon index, were deemed to be normally distributed. Variables that were non-parametric were appropriately transformed to be parametric, depending on the skewness of the variable. The non-parametric values were used in the initial unadjusted analysis, and the transformed variables were used in the adjusted analysis. The Simpson index was transformed using reflect and square root transformations, observed species was log_10_ transformed. Normality of all transformed data was confirmed before adjusted analysis. Median and IQR are presented for these variables. For all other continuous variables results are presented as mean (Standard deviation (SD)) and categorical variables are presented as n, (%). Well-being scores are presented as mean (SD) of total scores, percentage score, and well-being categories for both early pregnancy (16 weeks) and late pregnancy (34 weeks). Unadjusted differences between infant microbial diversity outcomes were assessed using independent T Tests and Mann Whitney *U* Tests. Differences in all variables between well-being groups in early pregnancy were also determined. Initial differences in microbiome diversity based on breastfeeding at discharge and breastfeeding at 1 month postpartum were determine using independent *T* Tests and Mann Whitney *U* tests. Significant associations were further included in regression analysis. Confounders were determined *a priori*, and included maternal age, maternal early-pregnancy BMI, probiotic vs. placebo trial group, mode of delivery (vaginal vs. cesarean delivery), antibiotics during delivery. Models with a *p* < 0.05 were considered significant with 95% confidence intervals. Adjusted analysis results are presented in Supplementary Tables. Bejamini-Hochberg Correction was applied to the adjusted analyses (Benjamini and Hochberg, 1995).

## 3. Results

### 3.1. Baseline information

Of the 118 participants, 53% received the probiotic intervention, 87% had 3rd level education, the average age was 33.38 years (SD +/− 3.93), and average BMI was 25.09 kg/m^2^ (SD +/− 3.28). The average birthweight was 3,653.48 g (SD +/− 548.62 g) and 50% of infants were male (Table 1). There were no differences in these values between women based on trial regimen of placebo vs. probiotic during pregnancy, except for maternal age (34.10 years vs. 32.59 years, respectively, *p* = 0.037), and birthweight (3,749.76 g vs. 3,546.88 g, respectively, *p* = 0.044) (Supplementary Table S1).

**Table 1:** Total group demographics.

Outcome	*n*	Mean	SD
Intervention group (n, %)	118	65	52.5
Age at recruitment (years)	118	33.38	3.93
BMI (Kg/m^2^)	118	25.09	3.28
HP index*	118	5.48	−0.72, 13.87
Completed 3rd level (n, %)	117	102	87
Caucasian ethnicity (n, %)	118	114	96.61
Supplements in pregnancy (n, %)	117	112	96
No alcohol in pregnancy (n, %)	118	74	63
No smoking in pregnancy (n, %)	118	115	97
First child (n, %)	118	68	57
Gestational age (days)*	118	281.50	276, 286
Birthweight (g)	118	3,653.48	548.62
Cesarean delivery (n, %)	118	24	20
Antibiotics in labor (n, %)	118	41	34
Male infant (n, %)	118	59	50
Neonatal intensive care unit admission (n, %)	118	12	10
*Breastfeeding practices at discharge (n, %)*			
Exclusive breast milk	115	74	65
Mixed feeding (breastmilk and formula)	115	20	17
Formula	115	21	18
*Breastfeeding practices at 1 month (n, %)*			
Exclusive breast milk	115	56	48
Mixed feeding (breastmilk and formula)	115	25	22
Formula	115	34	30

Exclusive breast milk refers to breastmilk from either the breast or bottle. Continuous variables are presented as mean and standard deviation unless non-normally distributed in which case*, it is the median (25th, 75th centile). Categorical outcomes presented as n (%).

### 3.2. Well-being

**Figure 1:**
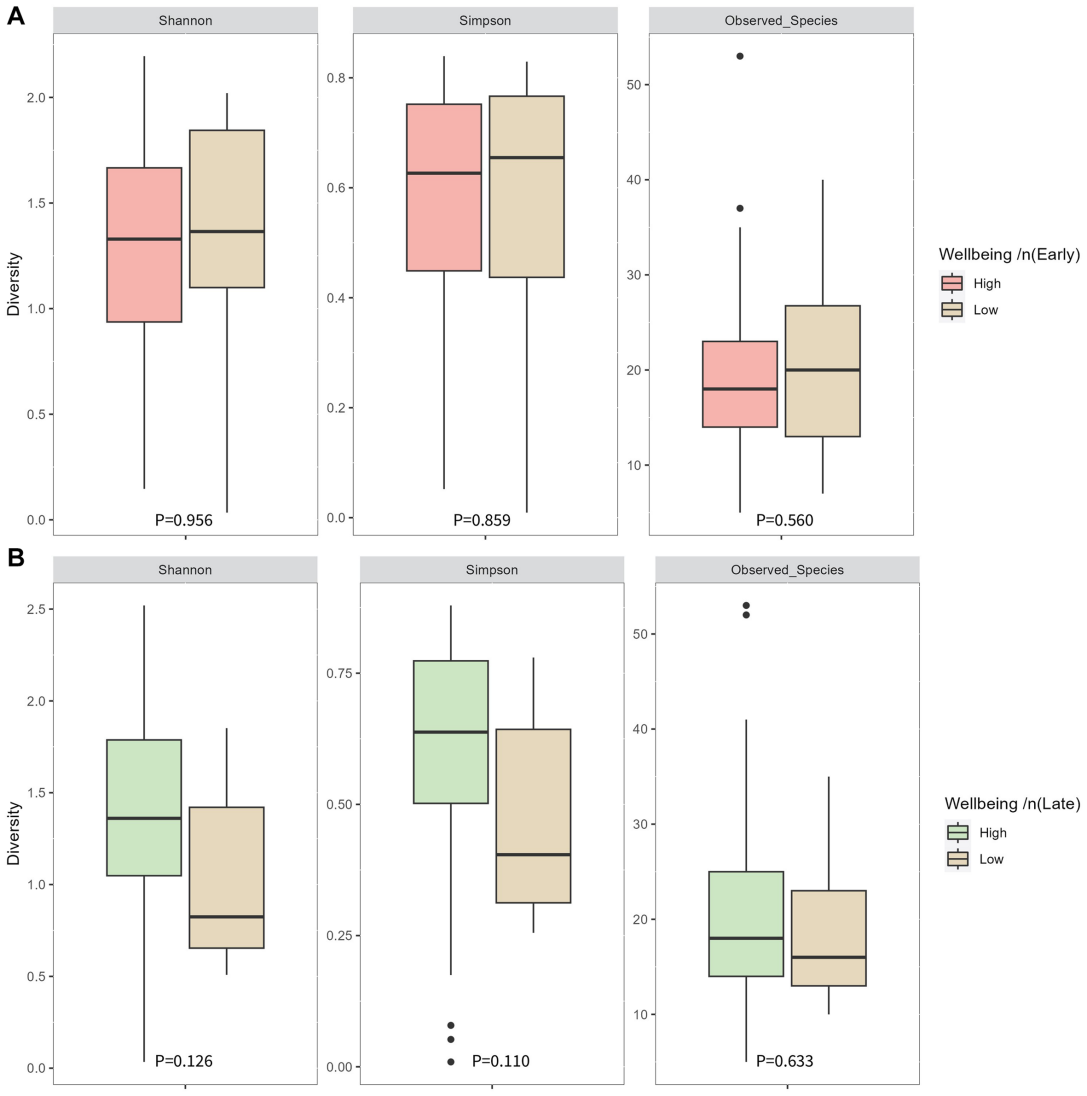
Infant microbial alpha diversity at 1 month of age compared between well-being groups in early pregnancy **(A)** and late pregnancy **(B)**. Early pregnancy well-being was measured at 16 weeks’ gestation. Late pregnancy well-being was measured at 34 weeks’ gestation. P-values were determined using independent sample T tests for the Shannon index and Mann-Whitney U tests for the Simpson index and observed species.

Cronbach alpha for the WHO-5 well-being score at 16 weeks’ gestation and 34 weeks’ gestation was 0.779 and 0.738, respectively, indicating the scale is reliable within our population at both timepoints. Average well-being scores were 17/25 or 66.5% in early pregnancy, with 87% (*n* = 97) of women having a high well-being score (>50%). In late pregnancy, average scores were 16/25 or 66.7% and 94% of women (*n* = 107) had a high well-being score (>50%) (Table 2). There were no significant associations in unadjusted analysis between early or late pregnancy well-being scores and all measures of infant microbial diversity (Figures 1A,B 2A,B). Therefore, no adjusted analysis was completed.

**Table 2:** Average well-being scores of the MicrobeMom Cohort in early and late pregnancy.

	Mean	SD
*Early pregnancy (n = 111)*		
Total score	16.71	3.54
Percentage score (%)	66.5	14.15
*Late pregnancy (n = 114)*		
Total score	16.68	3.00
Percentage score (%)	66.7	11.99
**Categorizations**	**N**	**%**
*Early pregnancy (n = 111)*		
High well-being (>50%)	97	87
Low well-being (≤50%)	14	13
*Late pregnancy (n = 114)*		
High well-being (>50%)	107	94
Low well-being (≤50%)	7	6

Well-being determined using WHO-5 well-being questionnaire. SD standard deviation. Early pregnancy defined as 16 weeks and late pregnancy defined as 34 weeks. Maximum total score is 25.

**Figure 2:**
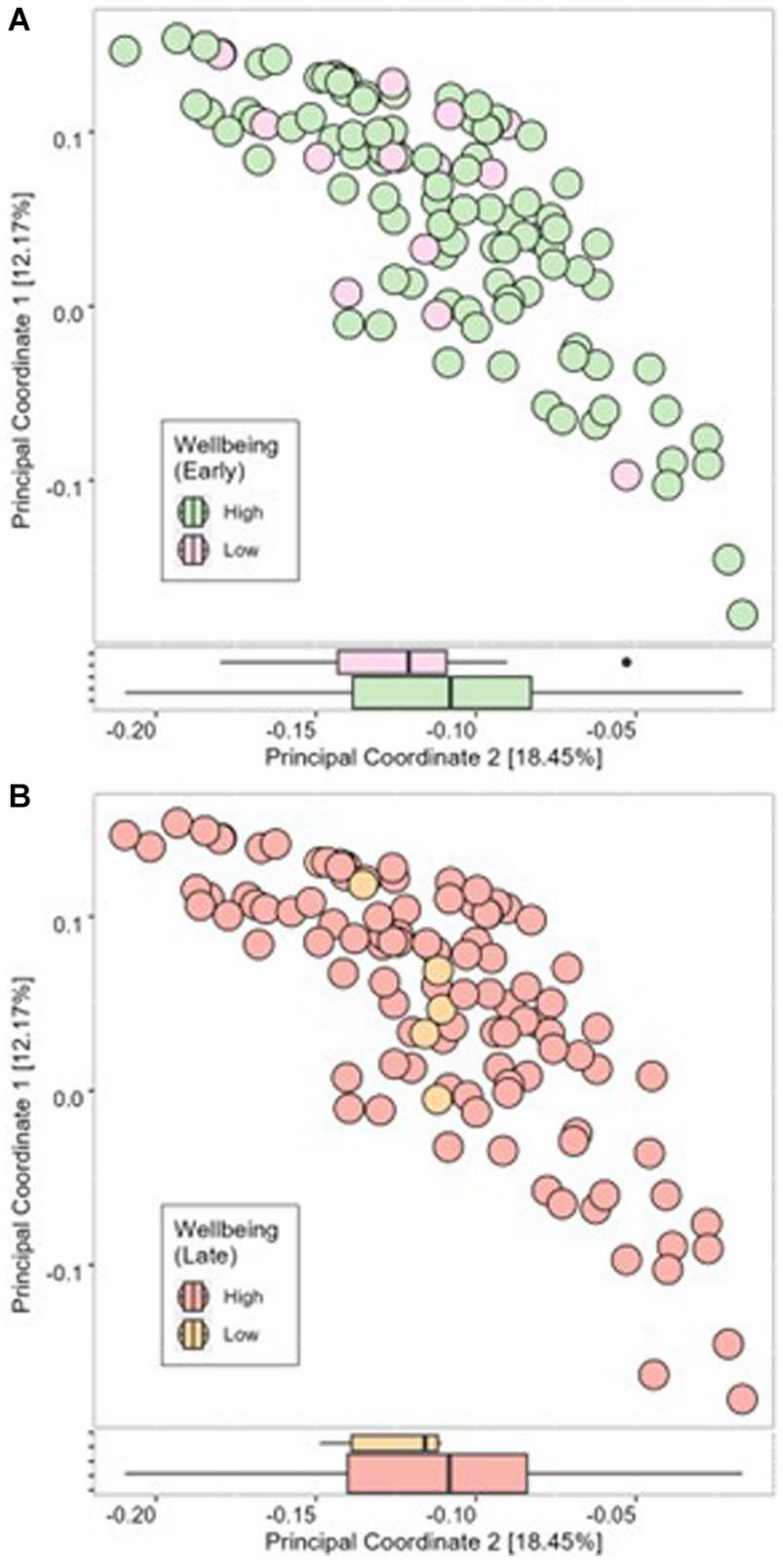
Beta diversity of infant microbial samples at 1 month postpartum as determined using Bray-Curtis dissimilarity measure compared between high well-being and low well-being in early pregnancy **(A)** and late pregnancy **(B)**.

### 3.3. Breastfeeding

**Figure 3:**
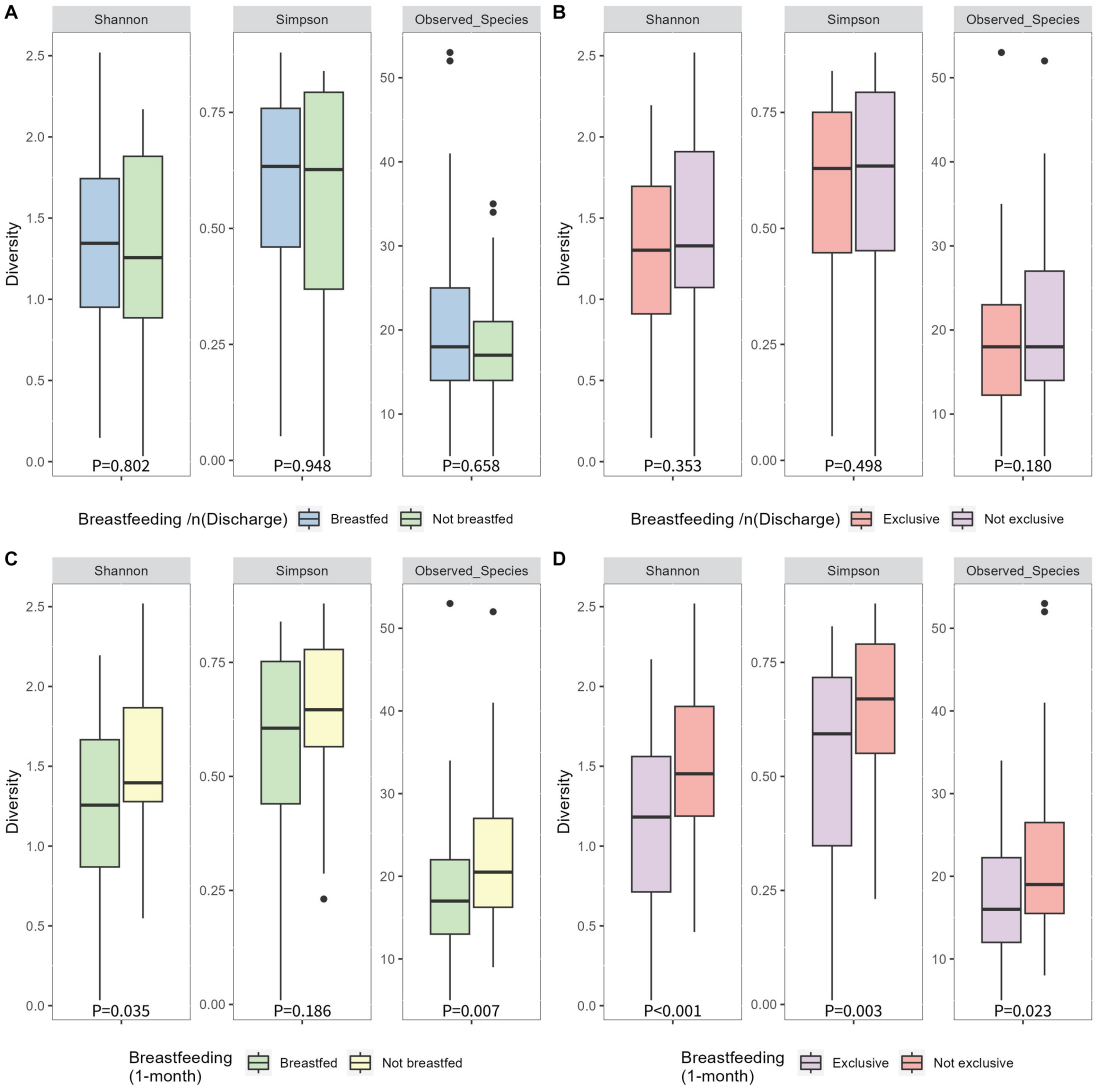
Alpha diversity of infant 1-month postpartum microbial samples as determined by Simpson, Shannon and observed species indices compared between breastfeeding habits of any breastfeeding at hospital discharge **(A)**, exlcusive breastfeeding at hospital discharge **(B)**, any breastfeeding at 1 month postpartum **(C)** and exclusive breastfeeding at 1 month postpartum **(D)**. P-values were determined using independent sample T tests for the shannon index and Mann-Whitney U tests for the simpson index and observed species.

**Figure 4:**
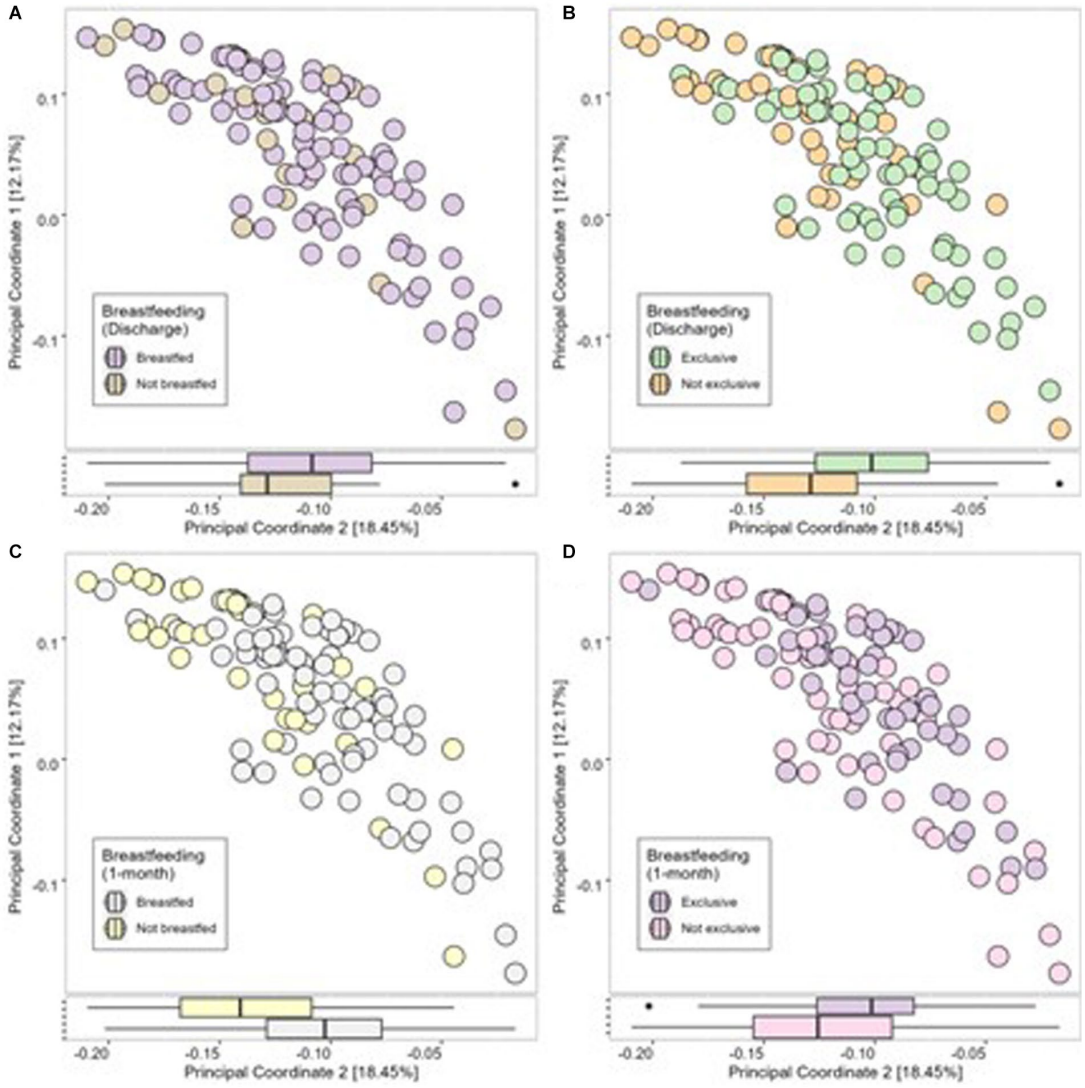
Infant microbial Beta Diversity as measured using Bray-Curtis dissimilarity measure compared between breastfeeding practices of any breastfeeding at discharge **(A)**, exclusive breastfeeding at discharge **(B)**, any breastfeeding at 1 month **(C)** and exclusive breastfeeding at 1 month **(D)**.

At discharge, 65% of mothers exclusively breastfed, 17% were feeding using a combination of breastmilk and formula milk, and 18% were using only formula milk. At 1 month postpartum, 48% of mothers were exclusively breastfeeding, 22% were using a combination of breastmilk and formula milk, and 30% were using formula milk only (Table 1). Therefore, 21 infants (18%) did not receive breastmilk at discharge, and 34 infants (30%) were not receiving breastmilk at 1 month postpartum. No significant differences were found between infant’s 1-month Shannon index, Simpson index or number of observed species (alpha diversity; Figures 3AB) or the Bray-Curtis measure of dissimilarity (beta diversity; Figure 4A) and any breastfeeding upon hospital discharge. Exclusive breastfeeding upon hospital discharge was negatively associated with infant beta diversity as per the Bray–Curtis dissimilarity measure at 1 month (Figure 4B). This significance remained in adjusted analysis (0.254, 95% CI 0.006, 0.038) (Supplementary Table S2). Any breastfeeding at 1 month postpartum was negatively associated with Shannon index, Simpson index and number of observed species (alpha diversity; Figure 3C) and the Bray-Curtis measure of dissimilarity (beta diversity; Figure 4C). Exclusive breastfeeding at 1 month postpartum was negatively associated with all measures of infant diversity at 1 month postpartum (Figures 3D 4D). In adjusted analysis, any breastfeeding at 1 month postpartum remained associated with alpha diversity (Shannon index; −0.241, 95% CI 0.498, −0.060 and observed species; −0.315, 95% CI −0.207, −0.060) and beta diversity (PC2; 0.319, 95% CI 0.013, 0.045). Exclusive breastfeeding remained associated with alpha diversity (Shannon index; −0.364, 95% CI −0.573, −0.194, Simpson index; 0.339, 95% CI 0.027, 0.091 and observed species; −0.271, 95% CI −0.172, −0.037) (Supplementary Table S3). Following application of the Benjamini-Hochberg Correction, exclusive breastfeeding at 1 month postpartum remained significantly associated with all metrics of alpha diversity. Any breastfeeding at 1 month postpartum remained associated with observed species and beta diversity (PC2) (Supplementary Table S3).

## 4. Discussion

This work examined the relationship between maternal mental well-being and offspring gut microbial diversity. Maternal prenatal well-being scores were not associated with infant microbial diversity at 1 month postpartum. Positive breastfeeding habits at discharge and 1 month postpartum was associated with lower infant gut microbial diversity at 1 month postpartum.

No relationship was found between early or late pregnancy maternal well-being and offspring microbiome diversity. A significant association between prenatal well-being and infant microbial diversity was hypothesized considering previous literature. Prenatal stress has previously been associated with higher abundance of bacterial strains known to contain pathogens and lower abundance of the more beneficial bacterial strains (Zijlmans et al., 2015). Additionally it was previously noted that maternal psychological distress during pregnancy was associated with the offspring gut bacterial families, but no association was found with microbial diversity (Naudé et al., 2020). This study used the Self-Reporting Questionnaire for determining psychological distress, and the Beck Depression Inventory was employed for depressive symptoms, measured in late pregnancy (28 weeks’ gestation). Similar to our own findings no relationship was found with maternal mental health factors and infant gut health. The relationship between maternal mental health factors during pregnancy and offspring microbiome is equivocal, requiring further research.

Despite the lack of associations found in our work to suggest a relationship between maternal prenatal well-being and offspring microbial diversity in infancy, maternal well-being scores remains an important predictor of other health outcomes for infants (Jairaj et al., 2019; O'Leary et al., 2019). The WHO-5 index is a clinically pragmatic tool that could easily be incorporated into routine antenatal care. The benefit of the WHO-5 index as opposed to other questionnaires surrounding mental health, is that it is short and easy to answer for pregnant women, it is easily interpreted by healthcare professionals and can cast a wider net in capturing those that may need intervention early in their pregnancy. There are numerous factors to consider when deciding upon a tool for use in clinical settings; the psychometric testing ability is not the sole consideration. Hermanns et al. propose that a screening tool should be short for use in a demanding clinical setting, and should provide simple, acceptable intuitive self-assessment (Hermanns et al., 2013). Furthermore, the WHO-5 index uses positively phrased questions which improves acceptability among patients (Hermanns et al., 2006).

We found compelling evidence that continuous breastfeeding may alter offspring gut microbiome. Exclusive breastfeeding at discharge from hospital was associated with some measures of infant microbial diversity, as was any breastfeeding and exclusive breastfeeding at 1 month postpartum. The greater extent of significant associations at 1 month postpartum compared to breastfeeding at hospital discharge suggests continued breastfeeding is important for infant health. The inverse associations between breastfeeding habits, alpha diversity and observed species may potentially be beneficial for the infant. It is plausible to suggest that a lower microbial diversity in infancy is preferable for infant health (van den Elsen et al., 2019; Yao et al., 2021). In a previous study, positive breastfeeding habits were associated with lower infant diversity; it was hypothesized that this may allow prioritization for *Bacteroides* colonization (Ma et al., 2020). It is known that the neonatal gut is predominantly occupied by species belonging to the *Bacteroides* genus, as these species are capable of metabolizing human milk oligosaccharides (Browne et al., 2022). This suggests that early neonatal gut health may not be demonstrated in a greater diversity of bacteria, but a greater abundance of specific *Bacteroides* such as the *Bifidobacterium* strains, though this requires more research. It was previously suggested that the introduction of solid foods alters infant gut microbiota, but this association was modified by prolonged breastfeeding habits (Differding et al., 2020). Furthermore, the KOALA Birth Cohort study noted that longer periods of breastfeeding improved microbiome composition at 6–9 years of age (Zhong et al., 2019). Such findings demonstrate the necessity for breastfeeding promotion. Considering the evidence of previous research and of our results, it appears that a lower diversity is preferred at this life stage, and a lower diversity is promoted by higher well-being and by prolonged breastfeeding habits. Further investigations are essential to confirm such findings.

This study is strengthened by several factors. The study was designed to model infant microbiome colonization and diversity using metagenomic shotgun sequencing, which is preferable to the typically used 16S rRNA sequencing methods as it is more detailed in its sequencing (Frankel et al., 2017). We examined both exclusive breastfeeding and any exposure to breastfeeding at two timepoints. We employed the WHO-5 well-being questionnaire, which is well-validated for the use in pregnant populations, to capture well-being and those at risk of depression (Topp et al., 2015), also measured at 2 timepoints. Furthermore, a paucity of research investigating outcomes of maternal health relating to well-being has been highlighted (Smith et al., 2014), thus highlighting the importance of this work. We are limited in this analysis as our study was not powered to find associations between maternal exposures and infant gut microbial diversity; the study was designed to determine the transfer of the probiotic from mother to infant. Therefore, we may have lost some correlations. We did not have longitudinal data on breastfeeding duration which may be related also to offspring microbiome. Furthermore, it was not possible to control for all early life factors that may impact offspring gut microbiome development, although efforts were made to adjust for most important predictors of infant gut health including mode of delivery, maternal BMI, and antibiotics during delivery.

## 5. Conclusion

Individual microbiome assessment of infants at birth would allow early intervention to improve their gut health, offering infants the best start in life. Our findings suggest that prenatal maternal well-being may not be related to the infant microbiome, but breastfeeding habits may be important for microbial diversity in infancy. Further research is needed to replicate and confirm such findings.

## Data availability statement

The raw data supporting the conclusions of this article will be made available by the authors, without undue reservation. All raw sequencing data and annotated whole genomes will be uploaded to ENA (accession PRJE48251). The authors will make the relevant anonymised patient level data available on reasonable request.

## Ethics statement

The studies involving humans were approved by ethical approval was granted by the National Maternity Hospital Ethics Committee in February 2019 (EC 35.2015). Informed written consent was obtained from participants. The authors assert that all procedures contributing to this work comply with the ethical standards of the relevant national and institutional committees on human experimentation and with the Helsinki Declaration of 1975, as revised in 2008. The studies were conducted in accordance with the local legislation and institutional requirements. The participants provided their written informed consent to participate in this study.

## Author contributions

FM and PC: conceptualization, resources, and software. RM, CF, CY, SLK, PC, DV, and FM: data curation. CY, SLK, and FM: formal analysis. RM, DV, FM, and PC: funding acquisition and methodology. CF, RM, CY, CW, EL, DB, AG, and SC: investigation. RM, DB, CY, AG, DV, PC, and FM: project administration. DV, PC, and FM: supervision. CF, SLK, FM, and PC: validation. CY and SLK: visualization and writing—original draft. CY, SLK, RM, SC, AG, DB, CF, CW, EL, EM, DV, PC, and FM: writing—review and editing. All authors contributed to the article and approved the submitted version.

## Funding

This publication has emanated from research supported in part by a peer-reviewed research grant from Science Foundation Ireland under grant number 12/RC/2273 and 16/SP/3827, which included a research grant from PrecisionBiotics Group Ltd. The funder was not involved in the study design, analysis, interpretation of data, the writing of this article or the decision to submit it for publication.

## Conflict of interest

The author EM was employed by PrecisionBiotics Group Ltd.

The remaining authors declare that the research was conducted in the absence of any commercial or financial relationships that could be construed as a potential conflict of interest.

## Publisher’s note

All claims expressed in this article are solely those of the authors and do not necessarily represent those of their affiliated organizations, or those of the publisher, the editors and the reviewers. Any product that may be evaluated in this article, or claim that may be made by its manufacturer, is not guaranteed or endorsed by the publisher.
