# Supplementary material for: Maternal breastfeeding is associated with offspring microbiome diversity; a secondary analysis of the MicrobeMom randomized control trial
Source: Front Microbiol. 2023 Aug 31;14:1154114. doi: 10.3389/fmicb.2023.1154114 (PMC10502216; doi:10.3389/fmicb.2023.1154114)
Supplement: Supplementary file 1 [file Data_Sheet_1.PDF]

For Submission to Frontiers in Microbiology

Supplementary Data

MATERNAL BREASTFEEDING, BUT NOT WELL-BEING, IS ASSOCIATED WITH OFFSPRING MICROBIOME DIVERSITY; A SECONDARY ANALYSIS OF THE MICROBEMOM RANDOMISED CONTROL TRIAL

Table S1 Differences between participant demographics in the probiotic vs. placebo groups of the MicrobeMom Study.

Variable	Placebo Group (n=62)		Probiotic Group (n=56)		p
	**n**	**%**	**N**	**%**	
Completed 3rd level education	56	90	46	82	0.407
Caucasian Ethnicity	58	94	56	100	0.121
First Child	31	50	37	48	0.094
Delivery – SVD	52	84	42	75	0.259
ABX in Labour	20	32	20	36	0.702
Male Infant	34	55	25	45	0.357
NICU Admission	9	15	3	54	0.132
Breastfeeding Practices at discharge					
Exclusive	36	58	38	68	0.244
Any breastfeeding	49	79	45	80	0.810
Breastfeeding Practices at 1 month					
Exclusive	29	47	27	48	0.935
Any breastfeeding	42	68	40	71	0.684
	**Mean**	**SD**	**Mean**	**SD**	**p**
Age at recruitment (years)	34.10	3.64	32.59	4.12	0.037
Height (m)	1.65	0.07	1.65	0.08	0.572
Weight (Kg)	67.74	8.35	68.64	10.00	0.599
BMI (Kg/m^2)	24.87	3.08	25.33	3.51	0.446
Gestational age (days)	279.23	9.63	281.75	8.34	0.133
Birthweight (g)	3749.76	562.90	3546.88	516.58	0.044
Early Well-being Score	16.68	3.76	16.75	3.34	0.921
Late Well-being Score	16.37	3.14	17.07	2.81	0.213
	**Median**	**IQR**	**Median**	**IQR**	**P**
HP Index	5.48	-0.77, 13.91	5.62	-0.99, 14.00	0.899

P value determined using Chi Square of categorical variables and independent T-tests used for continuous variables (mean / SD reported). P<0.05 significance. For HP index; Mann Whitney U Test used for P value and median / 25th, 75th centile presented. SVD spontaneous vaginal delivery; ABX antibiotics; NICU neonatal intensive care unit; Early well-being 16 weeks; Late well-being 34 weeks; BMI body mass index; HP index Pobal Haase & Pratschke Deprivation Index

Table S2. Adjusted linear regression analysis of breastfeeding habits at discharge and outcomes of infant microbiome diversity

Models	B	p	95% Confidence interval	R^2 Adj.
Outcome – PC2				
Exclusive Breastfeeding	0.254	0.007	0.006, 0.038	0.153

Model is controlled for delivery mode (SVD vs LSCS), antibiotics at delivery, maternal age, maternal body mass index, and study group (probiotic vs. placebo). P< 0.05 significance.

Table S3. Adjusted linear regression analysis of breastfeeding habits at 1 month postpartum and outcomes of infant microbiome diversity

Models	B	p	95% Confidence interval	R^2 Adj.
Outcome – Shannon Diversity				
Any Breastfeeding	-0.241	0.013	-0.498, -0.060	0.042
Exclusive Breastfeeding***	-0.364	<0.001	-0.573, -0.194	0.117
Outcome – Simpson Diversity *				
Exclusive Breastfeeding***	0.339	<0.001	0.027, 0.091	0.084
Outcome – Observed Species **				
Any Breastfeeding***	-0.315	<0.001	-0.207, -0.060	0.229
Exclusive Breastfeeding***	-0.271	0.003	-0.172, -0.037	0.207
Outcome – PC2				
Any Breastfeeding***	0.319	<0.001	0.013, 0.045	0.236
Exclusive Breastfeeding	0.014	0.062	-0.001, 0.029	0.124

* Reflect and square root transformation applied due to skew of data. **log10 transformation applied due to skew of data. All models are controlled for delivery mode (SVD vs LSCS), antibiotics at delivery, maternal age, maternal pre-pregnancy BMI and study group (intervention vs. control). P< 0.05 significance. *** Remained significant following Benjamini Hochberg Correction.
